# Correction: Integrin-linked kinase-frizzled 7 interaction maintains cancer stem cells to drive platinum resistance in ovarian cancer

**DOI:** 10.1186/s13046-024-03102-y

**Published:** 2024-06-22

**Authors:** Rula Atwani, Rohit Pravin Nagare, Amber Rogers, Mayuri Prasad, Virginie Lazar, George Sandusky, Yan Tong, Fabrizio Pin, Salvatore Condello

**Affiliations:** 1 Department of Obstetrics and Gynecology, Indiana University School of Medicine, Indianapolis, IN 46202 USA; 2 Indiana University Melvin and Bren Simon Comprehensive Cancer Center, Indianapolis, IN 46202 USA; 3 Department of Pharmacology & Toxicology, Indiana University School of Medicine, Indianapolis, IN 46202 USA; 4 Department of Pathology and Laboratory Medicine, Indiana University School of Medicine, Indianapolis, IN 46202 USA; 5 Department of Biostatistics and Health Data Science, Indiana University School of Medicine, Indianapolis, IN 46202 USA; 6 Department of Anatomy, Cell Biology and Physiology, Indiana University School of Medicine, Indianapolis, IN 46202 USA

**Correction:** ***J Exp Clin Cancer Res*** **43, 156 (2024)**

10.1186/s13046-024-03083-y

Following publication of the original article [1], the authors identified an error in the author name of Rohit Pravin Nagare.

The incorrect author name is: Rohit Nagare

The correct author name is: Rohit Pravin Nagare

The author group has been updated above and the original article [1] has been corrected.
