# Supplementary material for: Integrin-linked kinase-frizzled 7 interaction maintains cancer stem cells to drive platinum resistance in ovarian cancer
Source: J Exp Clin Cancer Res. 2024 Jun 1;43:156. doi: 10.1186/s13046-024-03083-y (PMC11143768; doi:10.1186/s13046-024-03083-y)
Supplement: Supplementary file 1 — Supplementary Material 1. [file 13046_2024_3083_MOESM1_ESM.docx]

**Supplementary Information**

**
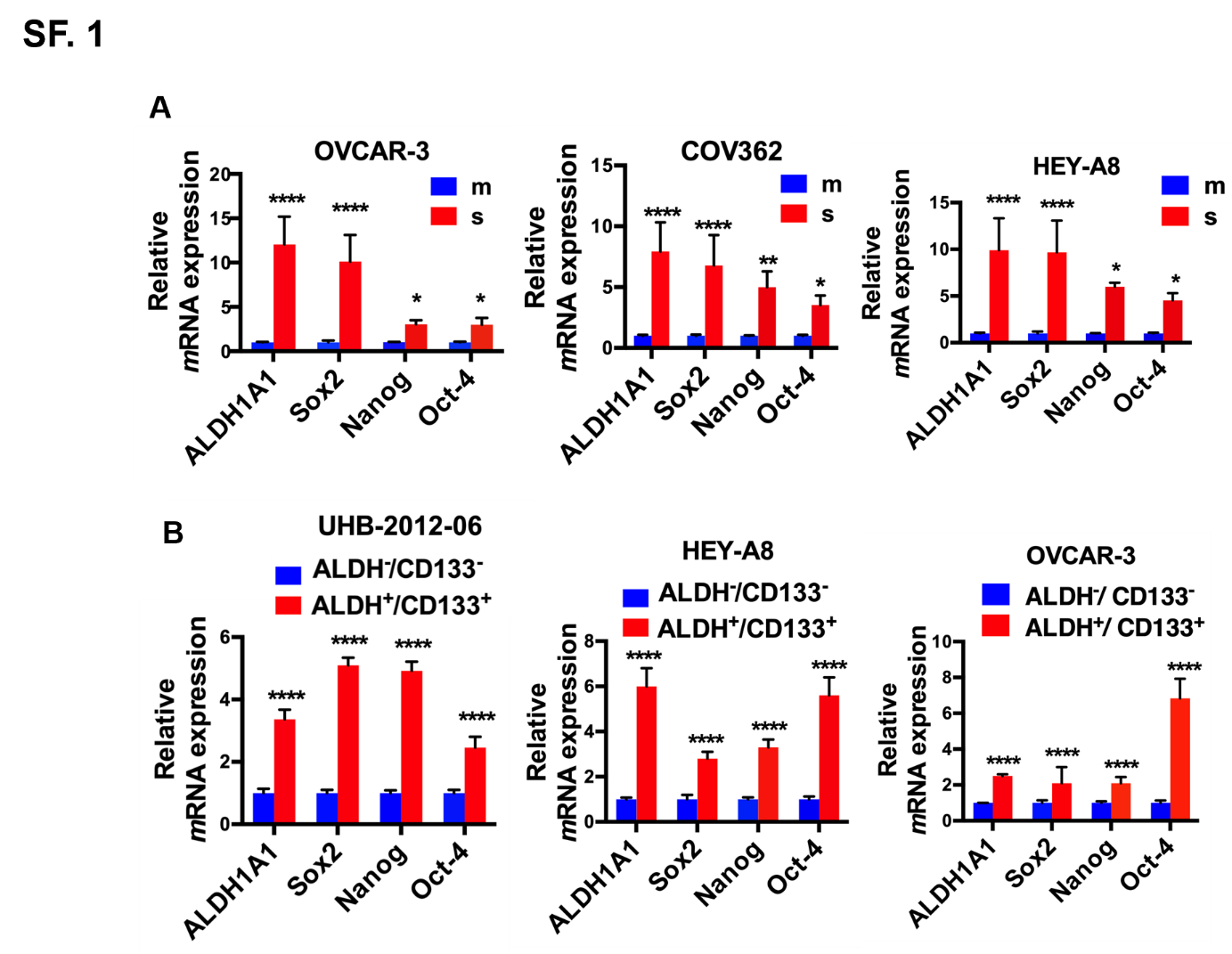
**

**Figure S1: ILK promotes the CSC phenotype.** **A.** *ALDH1A1*, *Sox-2*, *Nanog*, and *Oct-4 m*RNA levels measured by qRT-PCR in OVCAR-3, COV362, and HEY-A8 cells grown as monolayers (m) and spheroids (s) (N = 3; *P < 0.05, **P < 0.01, ****P < 0.0001). **B.** *ALDH1A1*, *Sox-2*, *Nanog*, and *Oct-4 m*RNA levels measured by qRT-PCR in ALDH^+^/CD133^+^ vs ALDH^-^/CD133^-^ isolated from UHB-2012-06, primary human OC cells, HEY-A8, and OVCAR-3 cells (N = 3; ****P < 0.0001).

**
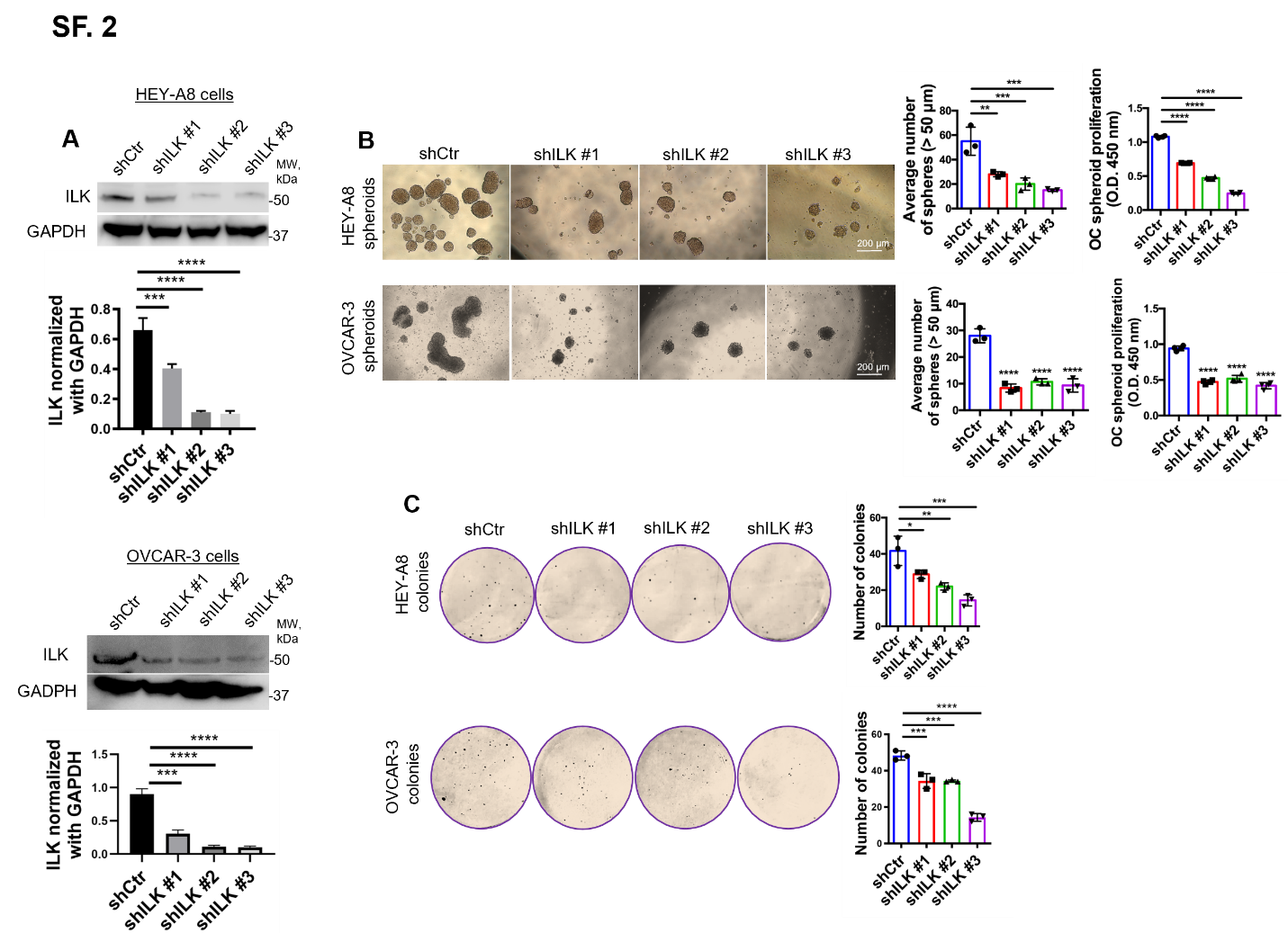
**

**Figure S2: ILK inhibition blocks OC spheroid proliferation, colony formation, and tumor initiation. A.** WB for ILK and GAPDH in shCtr and shILK (clones #1, #2, and #3) transduced HEY-A8 (upper panel) and OVCAR-3 cells (lower panel). **B.** Representative phase contrast images of shCtr and shILK (clones #1, #2, and #3) HEY-A8 and OVCAR-3 cells growing as spheroids (left panel). Average number of spheroid count (> 50 μm in diameter) (upper right panel) and CCK-8 quantification (lower right panel) of shCtr vs shILK (clones #1, #2, and #3) HEY-A8 and OVCAR-3 spheroids (N = 3; *P < 0.05, **P < 0.01, ***P < 0.001, ****P < 0.0001). **C.** Clonogenic assay of shCtr and shILK (clones #1, #2, and #3) HEY-A8 and OVCAR-3 cells (right panel). Representative images of single-cell clone proliferation, stained with crystal violet (left panel). Quantification of the results (left panel) (N = 3; *P < 0.05, **P < 0.01, ***P < 0.001P, ****P < 0.0001).


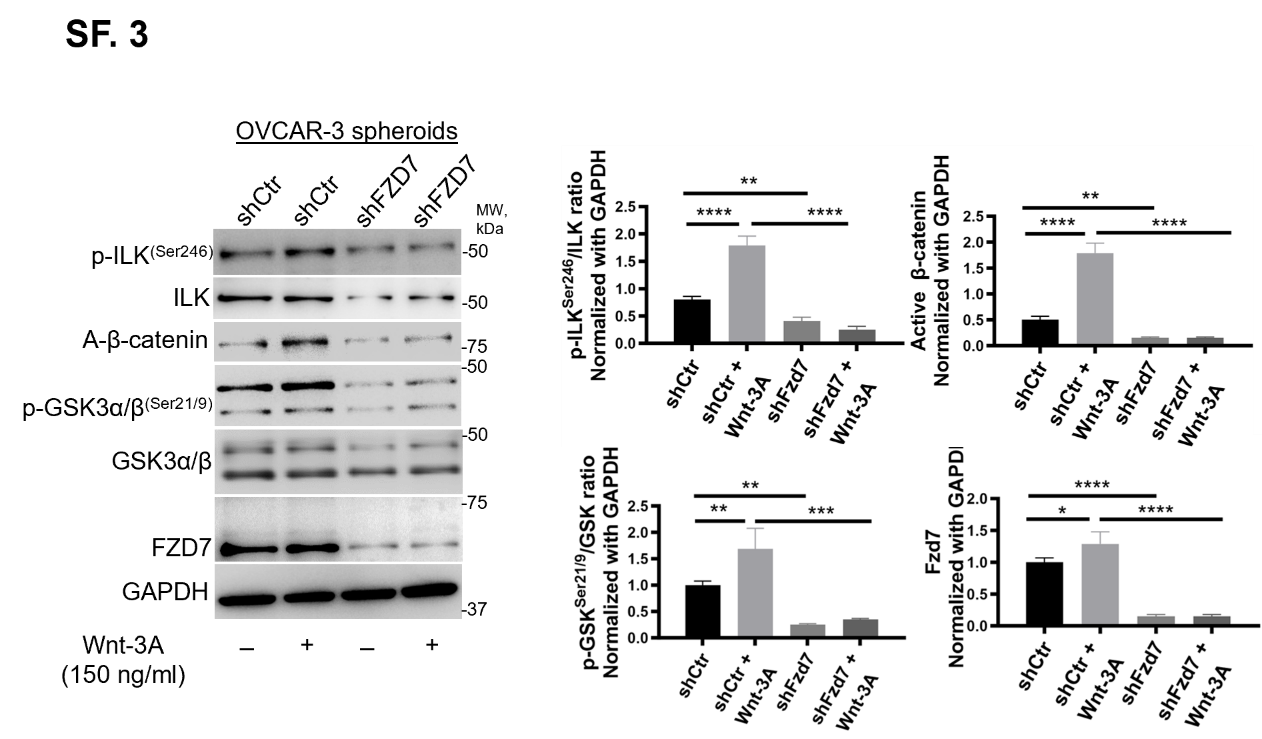


**Figure S3: ILK inhibition blocks the OCSC phenotype and regulates Fzd7 expression.** WB for A-β-catenin, p-GSK-3α/β^Ser21/9^, GSK-3α/β, Fzd7, p-ILK^Ser246^, ILK, and GAPDH in OVCAR-3 spheroids treated with or not with Wnt-3A (left panel). Densitometry quantifies A-β-catenin and Fzd7 expression levels and p-ILK^Ser246^/ILK and p-GSK-3α/β^Ser21/9^/GSK-3α/β ratio (right panel) (N = 3; *P < 0.05, **P < 0.01, ***P < 0.001, ****P < 0.0001).


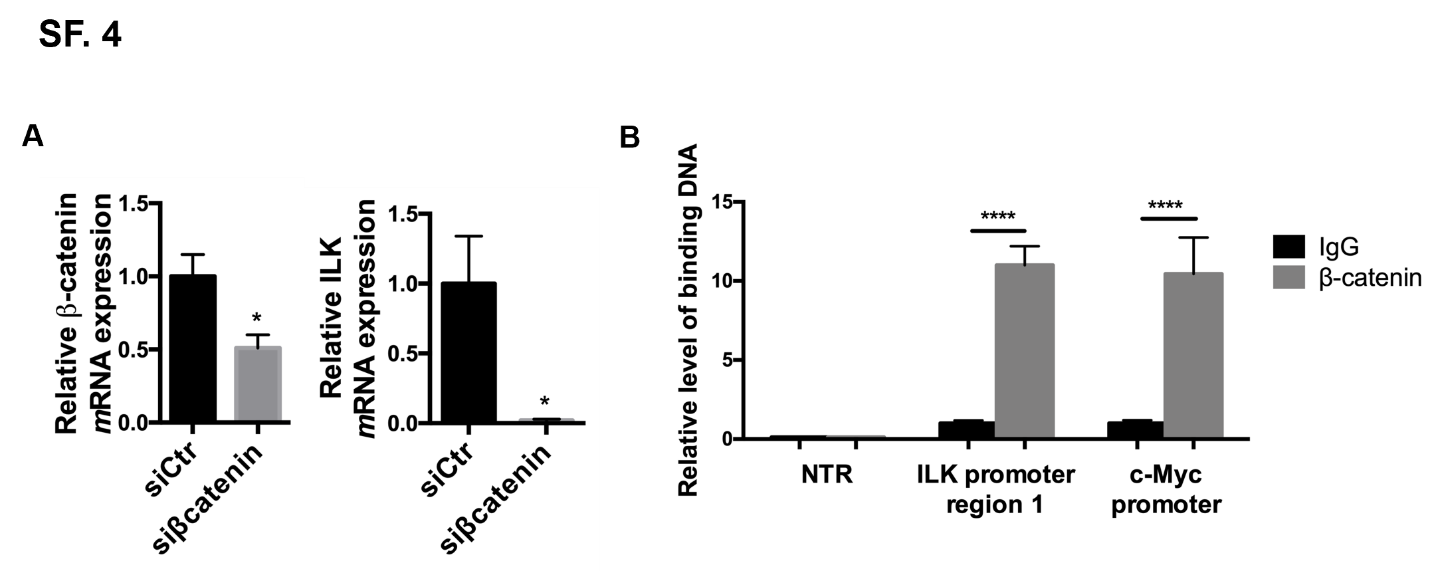


**Figure S4: ILK is a β-catenin target gene.** **A.** QRT-PCR for *β-catenin* and *ILK* *m*RNA expression levels in HEYA-8 cells transfected with scrambled or β-catenin targeting siRNA and grown as spheroids (N = 3; *P < 0.05). **B.** ChIP demonstrates that β-catenin/ TCF/LEF1 transcription complex binds the ILK promoter region. Immunoprecipitated chromatin with an antibody against β-catenin was used for quantitative PCR amplification. Primers flanking two predicted β-catenin/ TCF/LEF1-binding regions of the ILK promoter were used. Positive control was β-catenin antibody-immunoprecipitated chromatin amplified with primers for the *c-Myc* promoter, a known β-catenin target. Negative controls were chromatin immunoprecipitated with IgG and amplified with ILK promoter primers and chromatin immunoprecipitated with β-catenin antibody and amplified with primers to a region in the ILK promoter located upstream of the predicted β-catenin binding sites non-target region (NTR).


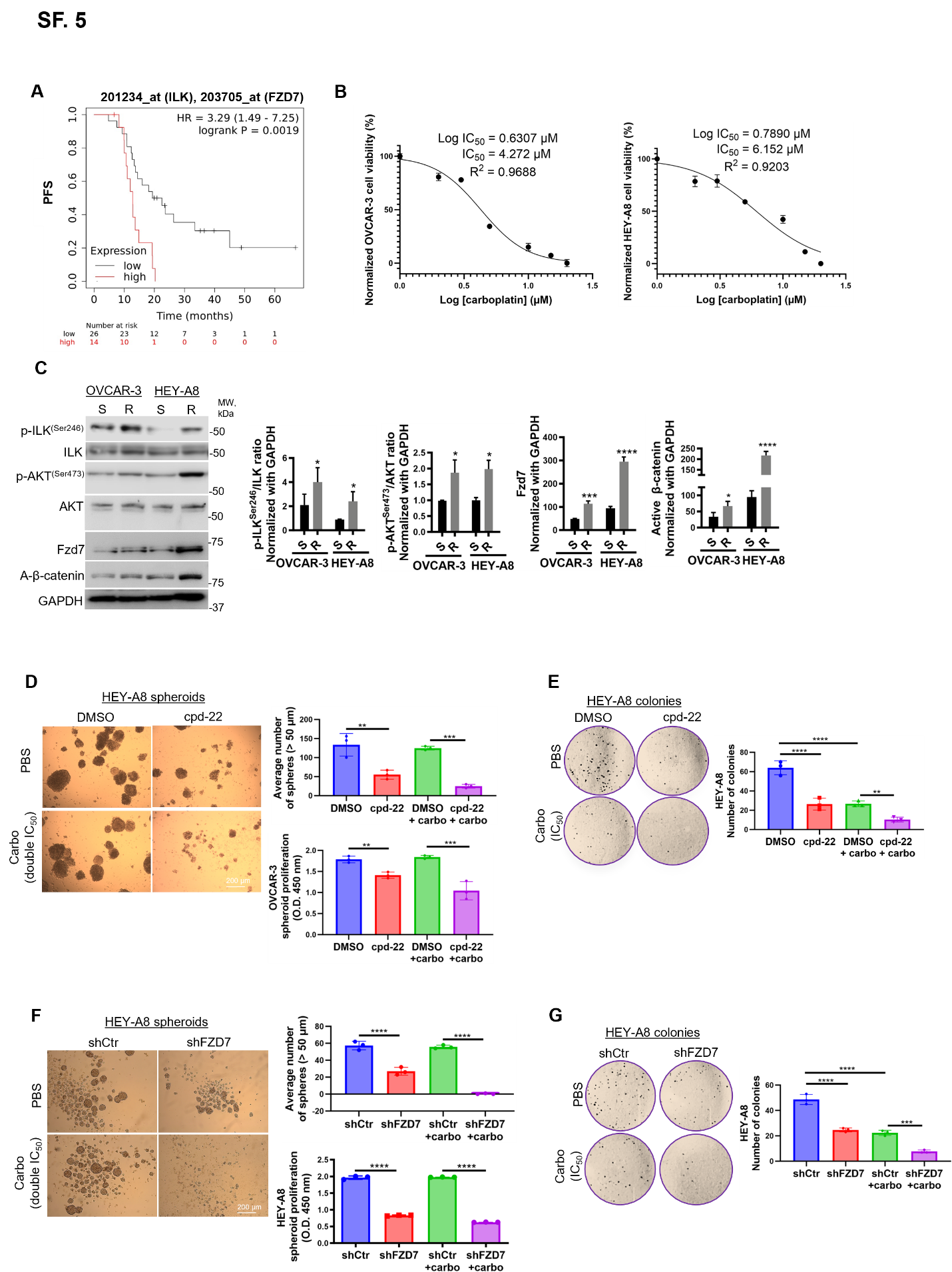


**Figure S5: Fzd7-KD and ILK inhibition sensitize to platinum treatment. A.** PFS curves for *ILK* in combination with *Fzd7* *m*RNA expression in HGSOC patients (stage: 3 + 4, grade: 3) following platinum/taxane therapy in the GSE14764 dataset using KM plotter (HR = 3.29, P = 0.0019). **B.** Survival curves for carboplatin tolerant OVCAR-3 (IC_50_ = 4.272 µM, R^2^ = 0.96; left panel) and HEY-A8 (IC_50_ = 6.152 µM, R^2^ = 0.92; right panel) cells. **C.** WB for Fzd7, p-ILK^Ser246^, ILK, p-AKT^Ser473^, AKT, and GAPDH in OVCAR-3 and HEY-A8 platinum (S) and platinum (R) (left panel). Densitometry quantifies Fzd7 expression and p-ILK^Ser246^/ILK and p-AKT^Ser473^/AKT ratio (right panel) (N = 3; *P < 0.05 and ***P < 0.001, ****P < 0.0001). **D.** Representative phase contrast images of HEY-A8 cells growing as spheroids and treated with DMSO, cpd-22, carboplatin (double IC_50_) or combination (left panel). Average number of spheroid count (> 50 μm in diameter) (upper-right panel) and CCK-8 quantification (lower-right panel) (N = 3; **P < 0.01, ****P < 0.0001). **E.** Clonogenic assay of HEY-A8 cells treated with DMSO, cpd-22, carboplatin (IC_50_) or combination. Representative images of single-cell clone proliferation, stained with crystal violet (left panel) and quantification (right panel) (N = 3; **P < 0.01 and ****P < 0.0001). **F.** Representative phase contrast images of shCtr vs shFzd7 (clone #2) HEY-A8 cells growing as spheroids and treated with carboplatin (double IC_50_) (right panel). Average number of spheroid count (> 50 μm in diameter) (upper-right panel) and CCK-8 quantification (lower-right panel) (N = 3; ****P < 0.0001). **G.** Clonogenic assay of shCtr vs shFzd7 (clone #2) HEY-A8 cells treated with carboplatin (IC_50_). Representative images of single-cell clone proliferation, stained with crystal violet (left panel) and quantification (right panel) (N=3; ***P < 0.001 and ****P < 0.0001).


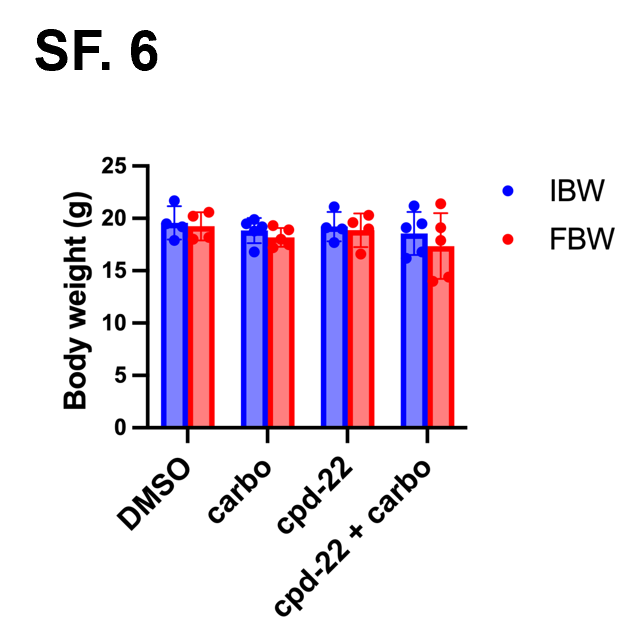


**Figure S6: Body weight changes in NSG mice.** Comparison between IBW and FBW in NSG mice treated with DMSO, carboplatin alone, cpd-22 alone, and/or combination (n = 5 per group). Data are shown as means ± s.e.m. Body weight was expressed in grams (g).

**Supplementary Table S1: List of antibodies used for WB, IF, IHC, PLA.**

| **Antibodies** | **Source** | **Catalog Number** |
| --- | --- | --- |
| AKT Rabbit | Cell Signaling | 9272 |
| ALDH1A1 (5A11) Mouse | Invitrogen | MA5-15692 |
| Bad (D24A9) Rabbit | Cell Signaling | 9239 |
| Caspase 3 (D3R6Y) Rabbit | Cell Signaling | 14220 |
| Cleaved Caspase 3 (Asp175) (5A1E) Rabbit | Cell Signaling | 9664 |
| CD133 Recombinant APC antibody [EPR20980-104] | Abcam | ab252129 |
| Frizzled 7 (4D9) Mouse | Santa Cruz Biotechnology | sc-293261 |
| GAPDH (6C5) Mouse | Invitrogen | AM4300 |
| GSK-3α/β (D75D3) Rabbit | Cell Signaling | 5676 |
| ILK (3A9) Mouse | Invitrogen | MA5-17099 |
| Non-phospho (Active) β-Catenin (Ser33/37/Thr41) Rabbit | Cell Signaling | 4270 |
| Phospho-AKT (Ser473) Rabbit | Cell Signaling | 9271 |
| Phospho-Bad (Ser136) (D25H8) Rabbit | Cell Signaling | 4366 |
| Phospho-GSK-3α/β (Ser21/9) Rabbit | Cell Signaling | 9331 |
| Phospho-ILK (Ser246) | Millipore-Sigma | AB1076 |
| Mouse anti-rabbit IgG-HRP | Santa Cruz Biotechnology | sc-2357 |
| Mouse control IgG | Santa Cruz Biotechnology | sc-515946 |
| Mouse IgG_1_ Alexa Fluor 488 | R&D Systems | IC002G |
| Mouse IgG_1_ APC | R&D Systems | IC002A |
| Mouse IgG2a, κ APC Isotype Control; Clone #G155-178 | BD Biosciences | 555576 |
| Rabbit control IgG | Santa Cruz Biotechnology | sc-2027 |
| Rabbit IgG HRP | R&D Systems | HAF008 |
| Rabbit IgG_1_ Alexa Fluor 488 | R&D Systems | IC1051G |
| Rabbit IgG Alexa Fluor 647 | R&D Systems | IC1051R |

**Supplementary Table S2: Primers used for Quantitative RT-PCR**

| Gene | 5' Primer | 3' Primer |
| --- | --- | --- |
| ALDH1A1 | AGGGGCAGCCATTTCTTCTCA | CACGGGCCTCCTCCACATT |
| β-catenin | GTTCGCCTTCACTATGGACTACC | GGACCCCTGCAGCTACTCTTT |
| c-Myc | CACCAGCAGCGACTCTGA | GATCCAGACTCTGACCTTTTGC |
| Fzd7 | GCCATCCCGCCGTGTCGTTCTCT | AGGGCGCGGTAGGGTAGGCAGTGG |
| ILK | TCAAACAGCTTAACTTCCTG | ATTTGGATGCGAGAAAATCC |
| Nanog | GATGCCTCACACGGAGACT | TTTGCGACACTCTTCTCTGC |
| Oct-4 | CTTCGCAAGCCCTCATTTC | GAGAAGGCGAAATCCGAAG |
| Sox2 | TGCTGCCTCTTTAAGACTAGGAC | CCTGGGGCTCAAACTTCTCT |
| 18S | ACCCGTTGAACCCCATTCGTGA | GCCTCACTAAACCATCCAATCGG |

**Supplementary Table S3: Primers used for ChIP assay**

| Gene | 5' Primer | 3' Primer |
| --- | --- | --- |
| ILK f1/r1 | CAGCGCTCCTCTGGAAGTC | GCCTGGCTAAACTCGATGGT |
| ILK upstream f/r | CATGCTCTCTTGTCCCAGGG | GACGTTGGGTCAGAGAGAGT |
| c-Myc | GTGAATACACGTTTGCGGGTTAC | AGAGACCCTTGTGAAAAAAACCG |

| **Tumor grade** | H-score < median | H-score > median | N |
| --- | --- | --- | --- |
| Serous low grade (stage IB-IIB) (FIGO) | 3 | 0 | 3 |
| Serous high grade (stage IIIC) (FIGO) | 2 | 4 | 6 |
| high grade (stage IV) (FIGO) | 1 | 5 | 6 |
| Metastasis | 8 | 17 | 25 |
| Normal ovary | 13 | 0 | 13 |
| Normal fallopian tube | 5 | 0 | 5 |
| All | 32 | 26 | 58 |
| Controls/Blanks |  |  | 8 |
| Missing/Torn/Rim/Artifact |  |  | 17 |
| Fat |  |  | 1 |
| SM Muscle |  |  | 2 |
| Necrosis |  |  | 1 |
| Stroma |  |  | 1 |
| Total Cores |  |  | 88 |

**Supplementary Table S4: p-ILK^Ser246^ expression in high grade serous ovarian tumors**

(Median H-score = 0.137).

**Supplementary Table S5:** Expression levels of stemness associated genes in cpd-22 compared to control (DMSO) treated OVCAR-3 cells grown as spheroids fold-changes (≥2.0, n = 3) of downregulated genes.

| RefSeq ID | Symbol | cpd-22 vs DMSO |
| --- | --- | --- |
| NM_000051 | *ATXN1* | -15.05755575 |
| NM_000332 | *AXL* | -8.674227799 |
| NM_002838 | *SAV1* | -8.214037492 |
| NM_032682 | *FZD7* | -6.374486512 |
| NM_002211 | *JAG1* | -6.263738689 |
| NM_030751 | *ZEB2* | -5.53899926 |
| NM_004827 | *ALCAM* | -5.50737858 |
| NM_000116 | *TGFBR1* | -5.067133595 |
| NM_001699 | *BMI1* | -4.757016711 |
| NM_000210 | *ITGB1* | -4.689551975 |
| NM_015238 | *YAP1* | -4.328181919 |
| NM_001556 | *CXCL8* | -4.320010749 |
| NM_000689 | *ATM* | -3.853742897 |
| NM_001627 | *ALDH1A1* | -3.802971363 |
| NM_000930 | *PLAUR* | -3.747200283 |
| NM_002093 | *HDAC1* | -3.641394425 |
| NM_000610 | *CHEK1* | -3.615716631 |
| NM_002051 | *GSK3B* | -3.607434005 |
| NM_001018016 | *MYC* | -3.55551124 |
| NM_001775 | *CD44* | -3.18734294 |
| NM_017617 | *NOTCH2* | -3.183730865 |
| NM_000584 | *ITGA2* | -3.144682918 |
| NM_021818 | *SIRT1* | -3.012940973 |
| NM_002165 | *IKBKB* | -2.715820861 |
| NM_002701 | *PROM1* | -2.495132693 |
| NM_006106 | *ZEB1* | -2.424177898 |
| NM_005985 | *SOX2* | -2.345074826 |
| NM_000222 | *KITLG* | -2.265580339 |
| NM_005378 | *NANOG* | -2.192930996 |
| NM_019074 | *DNMT1* | -2.133446501 |

| **Tumor grade** | H-score < median | H-score > median | N |
| --- | --- | --- | --- |
| Serous low grade (stage IB-IIB) (FIGO) | 3 | 0 | 3 |
| Serous high grade (stage IIIC) (FIGO) | 2 | 4 | 6 |
| high grade (stage IV) (FIGO) | 2 | 5 | 7 |
| Metastasis | 10 | 15 | 25 |
| Normal ovary | 13 | 0 | 13 |
| Normal fallopian tube | 5 | 0 | 5 |
| All | 35 | 24 | 59 |
| Controls/Blanks |  |  | 8 |
| Missing/Torn/Rim/Artifact |  |  | 16 |
| Fat |  |  | 1 |
| SM Muscle |  |  | 2 |
| Necrosis |  |  | 1 |
| Stroma |  |  | 1 |
| Total Cores |  |  | 88 |

**Supplementary Table S6: Fzd7 expression in high grade serous ovarian tumors**

(Median H-score = 0.124).
